# Supplementary figures and images for: Antisense non-coding transcription represses the PHO5 model gene at the level of promoter chromatin structure
Source: PLoS Genet. 2022 Oct 10;18(10):e1010432. doi: 10.1371/journal.pgen.1010432 (PMC9584416; doi:10.1371/journal.pgen.1010432)

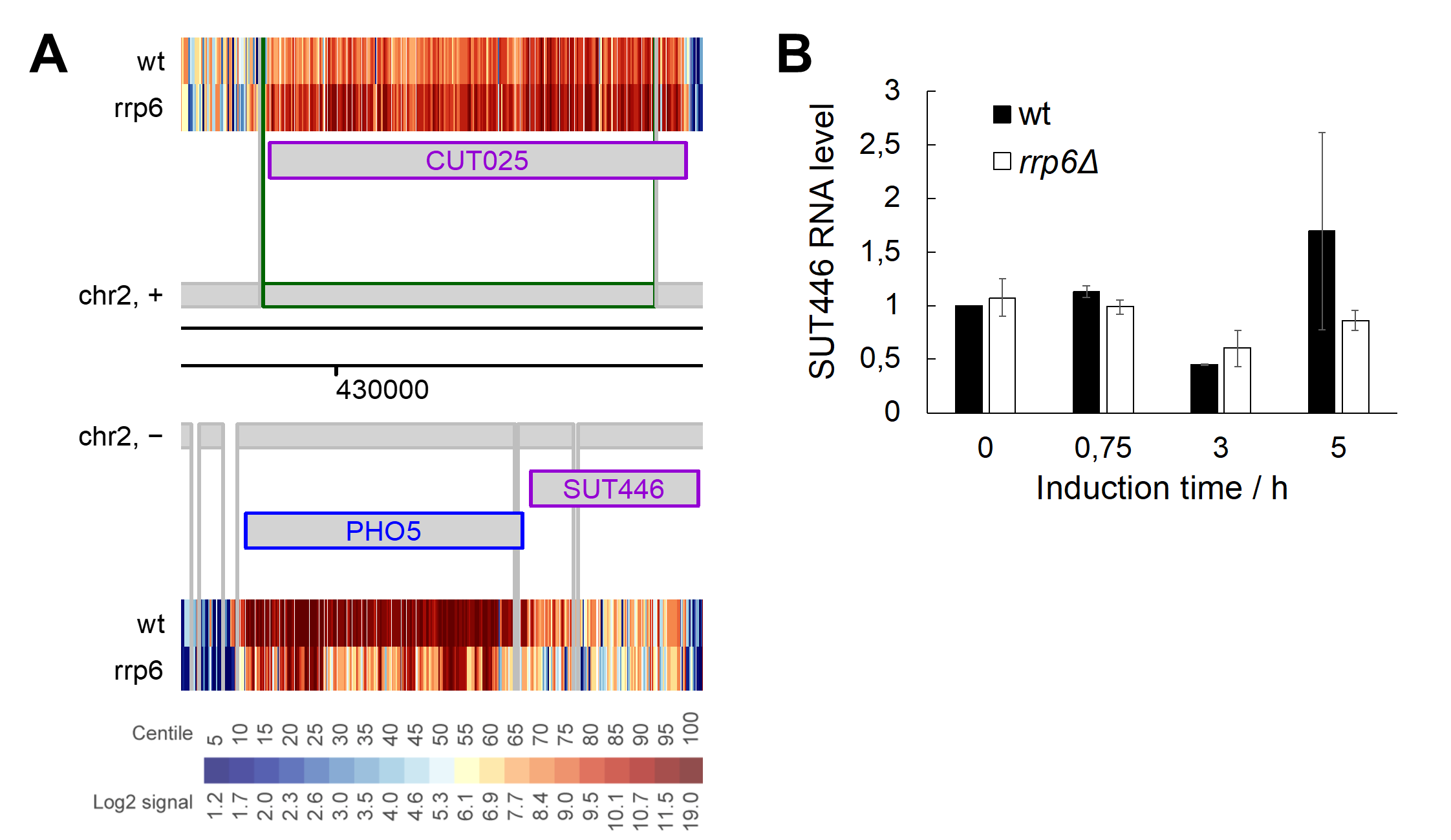

Supplement: S1 Fig — (A) A heatmap summarising tiling array expression data at the PHO5 gene locus in wild-type W101 (wt) and corresponding rrp6Δ cells. Data is from [36] and is visualized with the SGV Genomics Viewer [73]. (B) Levels of SUT446 in wild-type BMA41 (wt) and corresponding rrp6Δ mutant cells upon induction through phosphate starvation. RT-qPCR values were normalized to PMA1 RNA and expressed relative to transcript abundance in wild-type cells at repressive conditions (0 h of induction), which was set to 1. (TIFF) [file pgen.1010432.s001.tiff]

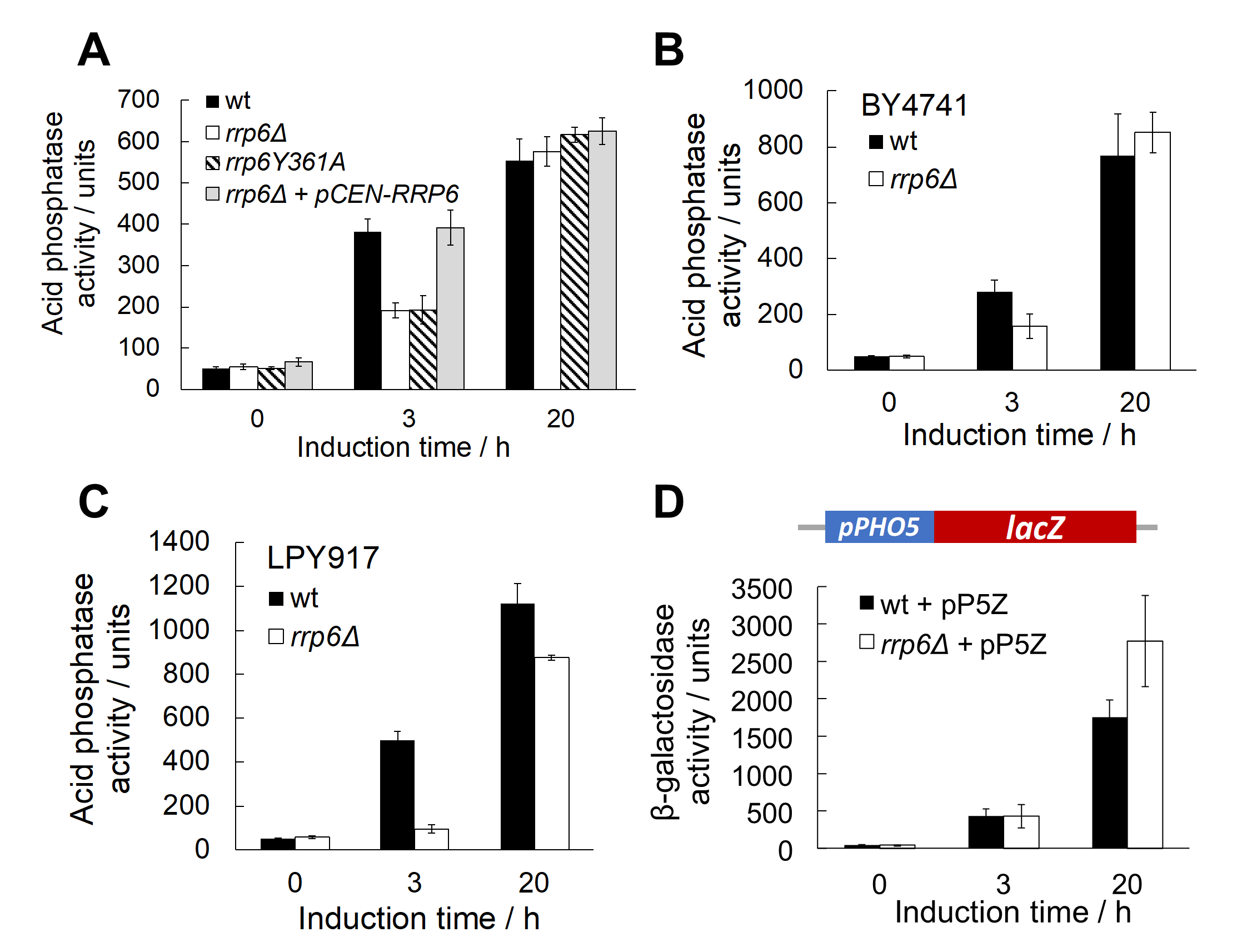

Supplement: S2 Fig — (A) Acid phosphatase induction kinetics in wild-type BMA41 (wt) and corresponding mutant cells upon induction through phosphate starvation. The strain rrp6Y361A carries a point mutation at the RRP6 genomic locus which abolishes exonuclease activity of Rrp6. Plasmid pCEN-RRP6 is a centromeric plasmid which carries the RRP6 gene under regulation of its native promoter. Reported values represent the means and standard deviations of three independent experiments (n = 3). (B) Same as (A), but for wild type and corresponding rrp6Δ mutant cells from the BY4741 genetic background. (C) Same as (A), but for wild type and corresponding rrp6Δ mutant cells from the LPY917 genetic background. (D) Beta-galactosidase induction kinetics in wild-type BMA41 (wt) and corresponding rrp6Δ cells transformed with a reporter plasmid pP5Z carrying the lacZ gene under the control of the PHO5 promoter upon induction through phosphate starvation. Reported values represent the means and standard deviations of three independent experiments (n = 3). (TIFF) [file pgen.1010432.s002.tiff]

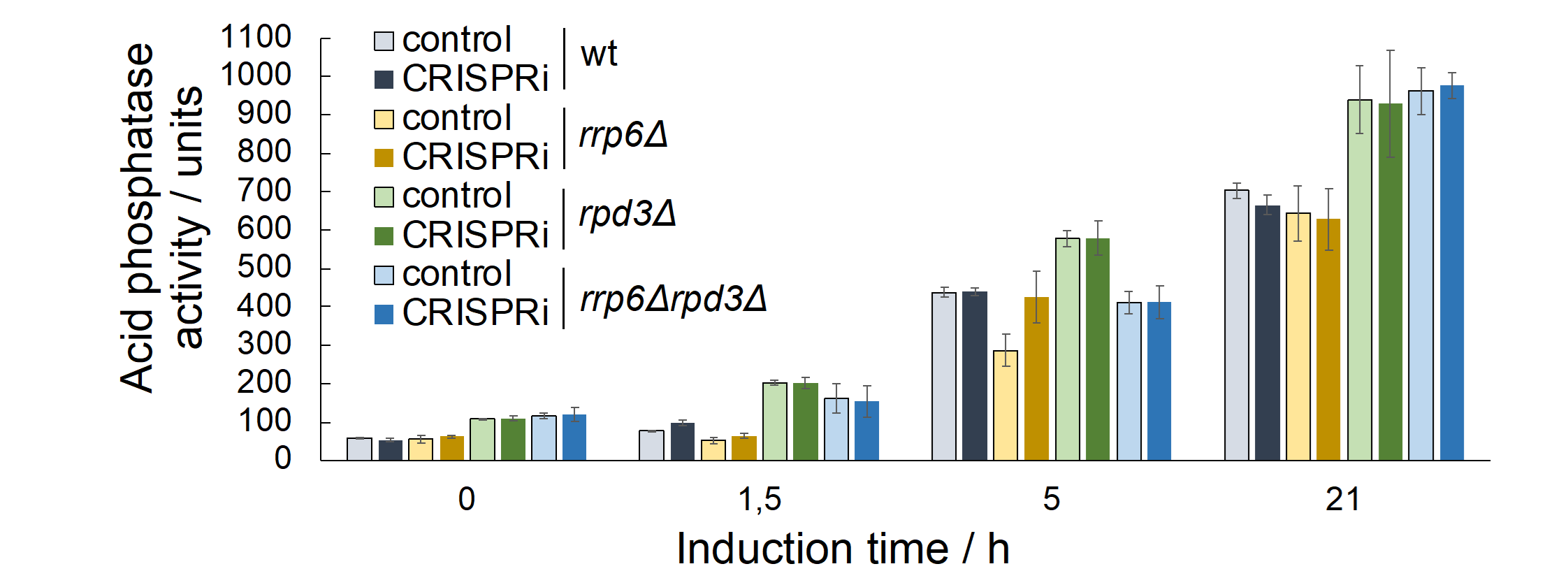

Supplement: S3 Fig — Acid phosphatase induction kinetics in wild-type BMA41 (wt) and corresponding deletion mutant cells for Rrp6 and Rpd3, with and without expression of the CRISPRi system which blocks PHO5 AS transcription, upon induction through phosphate starvation. Reported values represent the means and standard deviations of three independent experiments (n = 3). (TIFF) [file pgen.1010432.s003.tiff]

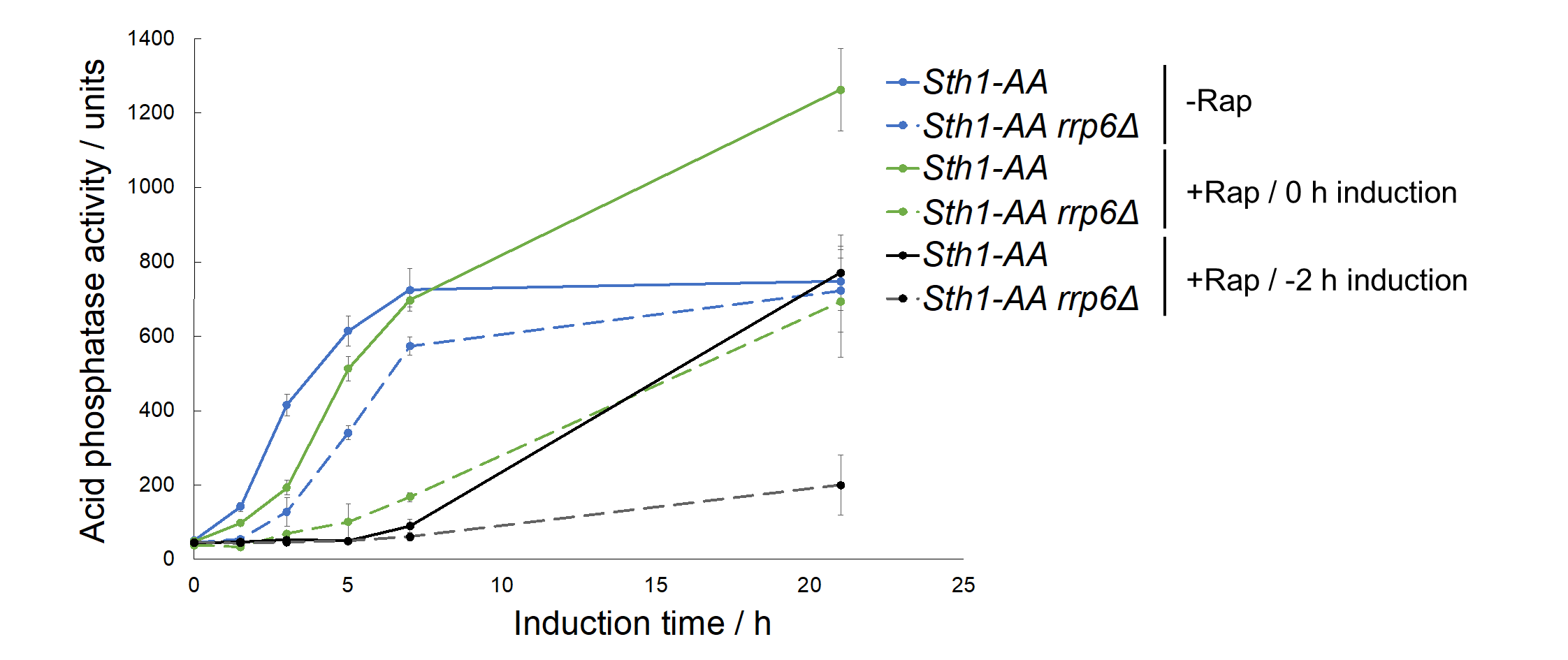

Supplement: S4 Fig — Acid phosphatase induction kinetics in Sth1-AA and the corresponding rrp6Δ cells upon induction through phosphate starvation without (-Rap) or with addition of rapamycin (+Rap) at indicated times. Reported values represent the means and standard deviations of three independent experiments (n = 3). (TIFF) [file pgen.1010432.s004.tiff]
