# Supplementary material for: Antisense non-coding transcription represses the PHO5 model gene at the level of promoter chromatin structure
Source: PLoS Genet. 2022 Oct 10;18(10):e1010432. doi: 10.1371/journal.pgen.1010432 (PMC9584416; doi:10.1371/journal.pgen.1010432)
Supplement: S1 Table — Table includes names of strains, their genotypes and sources. (PDF) [file pgen.1010432.s005.pdf]

Table S1. ***S. cerevisiae*** **strains**

Strain ID	Genotype	Source
BMA41 wild-type	*MATa ade2-1 ura3-1 leu2-3,112 his3-11,15 trp1Δ can1-100*	[1]
BMA41 *rrp6Δ*	BMA41 with *rrp6Δ::KanMX4*	[2]
BMA41 Rrp6-Y361A	BMA41 with *rrp6Y361A*	[3]
BMA41 *rrp47Δ*	BMA41 with *rrp47Δ::KanMX4*	[3]
BMA41 *trf4Δ*	BMA41 with *trf4Δ::KanMX4*	[3]
BMA41 *trf5Δ*	BMA41 with *trf5Δ::KanMX4*	[3]
BMA41 *mpp6Δ*	BMA41 with *mpp6Δ::KanMX4*	[3]
BMA41 *air1Δ*	BMA41 with *air1Δ::KanMX4*	[3]
BMA41 *air2Δ*	BMA41 with *air2Δ::KanMX4*	[3]
BMA41 *air1Δ air2Δ*	*MATa ade2-1 ura3-1 leu2-3,112 his3-11,15 trp1Δ can1-100 air1Δ::HIS3 air2Δ::KanMX4*	[2]
BMA41 *TEF1-PHO5AS*	BMA41 with *TEF1-PHO5AS::KanMX4*	This work
dis3Δ + pDis3	*MATa ade2-1 ura3-1 leu2-3,112 his3-11,15 trp1-1 can1-100 dis3Δ::KanMX4 [pBS3269-DIS3, LEU2]*	[3]
dis3Δ + pDis3-endo$^-$	*MATa ade2-1 ura3-1 leu2-3,112 his3-11,15 trp1-1 can1-100 dis3Δ::KanMX4 [pBS3278-dis3D171N, LEU2]*	[3]
dis3Δ + pDis3-exo$^-$	*MATa ade2-1 ura3-1 leu2-3,112 his3-11,15 trp1-1 can1-100 dis3Δ::KanMX4 [pBS3270-dis3D551N, LEU2]*	[3]
BY4741 wild-type	*MATa his3Δ1 leu2Δ0 met15Δ0 ura3Δ0*	[4]
BY4741 *rrp6Δ*	BY4741 with *rrp6Δ::KanMX4*	[5]
BY4741 *gcn5Δ*	BY4741 with *gcn5Δ::KanMX4*	EUROSCARF
BY4741 *gcn5Δ rrp6Δ*	BY4741 with *gcn5Δ::KanMX4 rrp6Δ::hph*	This work
LPY917 wild-type	*MATa ade2-101 his3Δ-200 leu2Δ1 trp1Δ1 lys2-801 TELadh4::URA3*	[6]
LPY917 *rrp6Δ*	LPY917 with *rrp6Δ::KanMX4*	This work
Nrd1-AA	*MATalpha tor1-1 fpr1::NAT RPL13A-2xFKB12::TRP1 Nrd1-FRB::kanMX6*	[7]
FSY1742 wild-type	*MATa ade2 his3 leu2 trp1 ura3*	[8]
FSY3117 *rrp6Δ*	FSY1742 with *rrp6Δ::KANr*	[8]
FSY3383 *rpd3Δ*	FSY1742 with *rpd3Δ::TRP1*	[7]
FSY3384 *rpd3Δ rrp6Δ*	FSY1742 with *rpd3Δ::TRP1 rrp6Δ::KANr*	[7]
Sth1-AA	*MATalpha tor1-1 fpr1::NAT RPL13A-2xFKB12::TRP1 Sth1-FRB::kanMX6*	[9]
Sth1-AA *gcn5Δ*	Sth1-AA with *gcn5Δ::SpHIS5*	This work
Sth1-AA *rrp6Δ*	Sth1-AA with *rrp6Δ::KanMX4*	This work
FSY6857	*MATa bar1Δ::hisG BrdU-Inc::HIS3*	This work
FSY5439	*MATα trp1::TRP1*	This work
FSY9286	*MATa bar1Δ::hisG BrdU-Inc::HIS3 pho5Δ::URA3*	This work

FSY9287	*MATα trp1::TRP1 pho5Δ::URA3*	This work
FSY9288	*MATa bar1Δ::hisG BrdU-Inc::HIS3 PHO5-Sense-Terminator*	This work
FSY9291	*MATα trp1::TRP1 PHO5-Antisense-Terminator*	This work

REFERENCES

1. Baudin-Baillieu A, Tollervey D, Cullin C, Lacroute F. Functional analysis of Rrp7p, an essential yeast protein involved in pre-rRNA processing and ribosome assembly. Mol Cell Biol. 1997;17: 5023–5032. doi:10.1128/mcb.17.9.5023

2. Mosrin-Huaman C, Honorine R, Rahmouni AR. Expression of bacterial Rho factor in yeast identifies new factors involved in the functional interplay between transcription and mRNP biogenesis. Mol Cell Biol. 2009;29: 4033–4044. doi:10.1128/MCB.00272-09

3. Stuparevic I, Mosrin-Huaman C, Hervouet-Coste N, Remenaric M, Rahmouni AR. Cotranscriptional recruitment of RNA exosome cofactors Rrp47p and Mpp6p and two distinct Trf-Air-Mtr4 polyadenylation (TRAMP) complexes assists the exonuclease Rrp6p in the targeting and degradation of an aberrant messenger ribonucleoprotein particle (mRNP) in yeast. J Biol Chem. 2013;288: 31816–31829. doi:10.1074/jbc.M113.491290

4. Brachmann CB, Davies A, Cost GJ, Caputo E, Li J, Hieter P, et al. Designer deletion strains derived from Saccharomyces cerevisiae S288C: A useful set of strains and plasmids for PCR-mediated gene disruption and other applications. Yeast. 1998;14: 115–132. doi:10.1002/(SICI)1097-0061(19980130)14:2<115::AID-YEA204>3.0.CO;2-2

5. Novačić A, Beauvais V, Oskomić M, Štrbac L, Dantec A Le, Rahmouni AR, et al. Yeast RNA exosome activity is necessary for maintaining cell wall stability through proper protein glycosylation. Mol Biol Cell. 2021;32: 363–375. doi:10.1091/mbc.e20-08-0544-t

6. Nislow C, Ray E, Pillus L. SET1, a yeast member of the Trithorax family, functions in transcriptional silencing and diverse cellular processes. Mol Biol Cell. 1997;8: 2421–2436. doi:10.1091/mbc.8.12.2421

7. Castelnuovo M, Zaugg JB, Guffanti E, Maffioletti A, Camblong J, Xu Z, et al. Role of histone modifications and early termination in pervasive transcription and antisense-mediated gene silencing in yeast. Nucleic Acids Res. 2014;42: 4348–4362. doi:10.1093/nar/gku100

8. Camblong J, Iglesias N, Fickentscher C, Dieppois G, Stutz F. Antisense RNA Stabilization Induces Transcriptional Gene Silencing via Histone Deacetylation in S. cerevisiae. Cell. 2007;131: 706–717. doi:10.1016/j.cell.2007.09.014

9. Gill JK, Maffioletti A, García-Molinero V, Stutz F, Soudet J. Fine Chromatin-Driven Mechanism of Transcription Interference by Antisense Noncoding Transcription. Cell Rep. 2020;31. doi:10.1016/j.celrep.2020.107612
