# Supplementary material for: Antisense non-coding transcription represses the PHO5 model gene at the level of promoter chromatin structure
Source: PLoS Genet. 2022 Oct 10;18(10):e1010432. doi: 10.1371/journal.pgen.1010432 (PMC9584416; doi:10.1371/journal.pgen.1010432)
Supplement: S2 Table — Table includes names and sequences of oligonucleotides used for strain construction, plasmid construction and RT-qPCR. (PDF) [file pgen.1010432.s006.pdf]

Table S2. Primers

Primer ID	Sequence (5'→3')	Description
RRP6-Kan1	GATAGACGAAATAGGAACAACAAACAGCT TATAAGCACCCAATAAGTGCGTT CCCGGCCAGCGACATGGAGGCCCAG	Deletion of *RRP6* (KanMX4 marker)
RRP6-Kan2	GCCCTTGGTCCATTACTATCGCTAGATGA TGGGTCGAATCTCCTTTTCCGAATCG ACAGCAGTATAGCGACCAGGCT	Deletion of *RRP6* (KanMX4 marker)
RRP6hph_fwd	GAAATAGGAACAACAAACAGCTTATAAGC ACCCAATAAGTGCGTTACGATCATTCAAG AGATCCCCG	Deletion of *RRP6* (hph marker)
RRP6hph_rev	AATTACCATAATTTATAAATAAAAAAATAC GCTTGTTTTACATAATACCGCCTTTGAGT GAGCTG	Deletion of *RRP6* (hph marker)
RRP6_fwd	CCCAAAAATATGAGGGCATCGG	Confirmation of *RRP6* deletion
RRP6_rev	AAAATGGTGTGCATGGGGGA	Confirmation of *RRP6* deletion
RRP6ORF_fwd	AATAACCCCAGTCACTACCC	Confirmation of *RRP6* deletion
RRP6ORF_rev	ACAACACCGAAAACCTTTCC	Confirmation of *RRP6* deletion
gcn5HIS_fwd	GTGAGCCGCCCAAAAGTCTTCAGTTAACT CAGGTTCGTATTCTACATTAGCCGCTAGG GATAACAGGGT	Deletion of *GCN5*
gcn5HIS_rev	ATTTATTTCTTCTTCGAAAGGAATAGTAGC GGAAAAGCTTCTTCTACGCAAAGAGCGC CCAATACGCAAA	Deletion of *GCN5*
gcn5HIScheck1_fwd	TGGTAAGGGAAGACCGTGAG	Confirmation of *GCN5* deletion
gcn5HIScheck1_rev	TCGTCTCGCCGTACTAAACA	Confirmation of *GCN5* deletion
gcn5HIScheck2_fwd	GACAATGCCGCAAAAGTCCA	Confirmation of *GCN5* deletion
gcn5HIScheck2_rev	CCCGACAAGTCAACTACGCT	Confirmation of *GCN5* deletion
TEF1PHO5AS_fwd	AAAATTTGGGTATTCGTATTTAGTTTCCAA TATTATTTAGTTATACAAAACGTACGCTGC AGGTCGAC	Insertion of *TEF1* promoter to drive *PHO5* AS transcription
TEF1PHO5AS_rev	ACTGGGACTGGAACACTACTCATTACAAC GCCAGTCTATTGAGACAATAGCATCGATG AATTCTCTGTCG	Insertion of *TEF1* promoter to drive *PHO5* AS transcription
TEF1PHO5AS_check1_fwd	TGGGCAACACTTTCCACAGA	Confirmation of *TEF1-PHO5AS* construct
TEF1PHO5AS_check1_rev	TCCTGACTGACTACAGGGATTGA	Confirmation of *TEF1-PHO5AS* construct
TEF1PHO5AS_check2_fwd	GGTGCCGGACCATACTACTC	Confirmation of *TEF1-PHO5AS* construct
TEF1PHO5AS_check2_rev	ACGGTCTTCAATTTCTCAAGTTTC	Confirmation of *TEF1-PHO5AS* construct
PMA1_fwd	CAATCTAATCACGGTGTCGACGACGAAG AC	RT-qPCR
PMA1_rev	GGCTTCCATAACGAATTGAATTGGACCG	RT-qPCR

SCR1_fwd	AACCGTCTTTCCTCCGTCGTAA	RT-qPCR
SCR1_rev	CTACCTTGCCGCACCAGACA	RT-qPCR
PHO5prom_qPCR_fwd	CACGTGGGACTAGCACAGAC	RT-qPCR
PHO5prom_qPCR_rev	TGCCTTGCCAAGTAAGGTGA	RT-qPCR
PHO5ORF_qPCR_fwd	ACACTCGTCAATTCAACGGC	RT-qPCR
PHO5ORF_qPCR_rev	GAGCATCCAGTGTATGGGTTCA	RT-qPCR
PHO5_5adjreg_fwd	CCTTTACCGTAATTTTCAATTGCTAA	ChIP qPCR
PHO5_5adjreg_rev	TCGCTTCTTCAACAGTGGTAAAAATA	ChIP qPCR
PHO5_UASp2_fwd	GAATAGGCAATCTCTAAATGAATCGA	ChIP qPCR
PHO5_UASp2_rev	GAAAACAGGGACCAGAATCATAAATT	ChIP qPCR
OFS2869	TTTATAGGTTAAGGATAGTAAAGGAATAC AGGTAAG	Plasmid construction
OFS2870	ACTCACAAATTAGATAATTATCCTATAAAT ATAACGTTTTTGAACAC	Plasmid construction
OFS2871	TAGGATAATTATCTAATTTGTGAGTTTAGT ATACATGC	Plasmid construction
OFS2872	TCCTTTACTATCCTTAACCTATAAAAATAG GCGTATCACGAG	Plasmid construction
OFS5084	AGAACAACAACAAATAGAGCAAGCAAATTCGA GATTACCAcagctgaagcttcgtacgc	Strain construction
OFS5085	TATTCGTATTTAGTTTCCAATATTATTTAGTTAT ACAAAAgcataggccactagtggatctg	Strain construction
OFS5086	CATGAGAATAAGAACAACAACAAATAGAGCAA GCAAATTCGAGATTAGTAATGAATTAAGTCTTG ATATATAACAATTAGCTTGATGTTTAAATCTGT TGTTTATTCAATTTTAGCCGC	Strain construction (Sense terminator Forward)
OFS5087	CGGCAAAATTTAGATAAAAAATTTGGG	Strain construction (Sense terminator Reverse)
OFS5088	GTTAGTATGGCTTCATCTCTCATGAGAATAAGA	Strain construction (Antisense terminator Forward)
OFS5089	AAAAATTTGGGTATTCGTATTTAGTTTCCAATA TTATTTAGTTATACAGTAATGAATTAAGTCTTG ATATATAACAATTAGCTTGCTATTGTCTCAATA GACTGGCGTTGTAATG	Strain construction (Antisense terminator Reverse)
OFS2522_PHO5_fwd	CTTGGGACTACGATGCCAAT	RT-qPCR
OFS2523_PHO5_rev	ACTTCAAATGCACACCACGA	RT-qPCR
OFS1717_SCR1_fwd	AACCGTCTTTCCTCCGTCGTAA	RT-qPCR
OFS1718_SCR1_rev	CTACCTTGCCGCACCAGACA	RT-qPCR
